# The characteristics of effective technology-enabled dementia education for health and social care practitioners: protocol for a mixed studies systematic review

**DOI:** 10.1186/s13643-019-1212-4

**Published:** 2019-12-06

**Authors:** Kevin Muirhead, Leah Macaden, Charlotte Clarke, Keith Smyth, Rob Polson, Chris O’Malley

**Affiliations:** 1 Department of Nursing and Midwifery, School of Health, Social Care & Life Sciences, University of the Highlands and Islands, Centre for Health Science, Old Perth Road, Inverness, IV2 3JH UK; 2 School of Health in Social Science, University of Edinburgh, Teviot Place, Edinburgh, EH8 9AG UK; 3 Learning and Teaching Academy, University of the Highlands and Islands, Ness Walk, Inverness, IV3 5SQ UK; 4 Highland Health Sciences Library, Centre for Health Science, Old Perth Road, Inverness, IV2 3JH UK

**Keywords:** Dementia, Dementia education, Technology-enabled learning, Systematic review

## Abstract

**Background:**

The global prevalence of people living with dementia is expected to increase exponentially and yet evidence suggests gaps in dementia-specific knowledge amongst practitioners. Evidence-based learning approaches can support educators and learners who are transitioning into new educational paradigms resulting from technological advances. Technology-enabled learning is increasingly being used in health care education and may be a feasible approach to dementia education.

**Methods:**

This protocol aims to describe the methodological and analytical approaches for undertaking a systematic review of the current evidence based on technology-enabled approaches to dementia education for health and social care practitioners. The design and methodology were informed by guidelines from the Preferred Reporting Items for Systematic Review and Meta-Analysis Protocols.

**Discussion:**

The evidence generated from a systematic review of the current evidence is intended to inform the design and implementation of technology-enabled dementia education programmes and to advance the current academic literature at a time of unprecedented demographic and technological transition.

**Trial registration:**

PROSPERO, CRD42018115378.

## Background

Evidence-based practices are widely accepted across health care disciplines [1, 2]. Implementing evidence-based teaching practices in health care settings avoids reliance on traditional methods including expert opinions that have not been established in systematic research [1]. Educators who are transitioning into new teaching paradigms to meet the expanded needs and learning styles of students can be supported by evidence-based approaches, including new pedagogy and the use of technology for learning [3]. This protocol describes the planned methodological and analytical approaches for undertaking a systematic review of the current evidence on technology-enabled dementia education (TEDE) for health and social care practitioners (HSCPs). The features of effective dementia education for HSCPs were identified in a recent and comprehensive systematic review [4]. To date, there do not appear to be any published systematic reviews on the characteristics and effectiveness of TEDE.

Dementia is a chronic, progressive syndrome in which there is disturbance of multiple higher cortical functions. Alzheimer’s disease, vascular dementia, dementia with Lewy bodies, and frontotemporal dementia are common subtypes although boundaries are indistinct and mixed forms co-exist [5]. The global prevalence of people living with dementia is 47 million and is predicted to rise to 135 million by 2050 [6]. Within the UK context, 850,000 people (one in 14 adults over the age of 65) are estimated to be living with dementia with the future prevalence predicted to mirror global trends [7]. Concern about the quality of care for people living with dementia has intensified the need for an appropriately educated workforce [8] with evidence suggesting gaps in dementia-specific knowledge amongst practitioners [9]. Furthermore, the undergraduate dementia education agenda is variable and dependent on the curricular priorities of Higher Education Institutions [10]. There is, therefore, a growing need for the wide dissemination of dementia education for those involved in meeting the care needs of people living with dementia [11].

TEDE is a dementia-specific form of technology-enabled learning (TEL). TEL is increasingly being adopted in medical and health care education as an effective approach compared to traditional learning for knowledge and skills acquisition [12]. In this protocol, TEL is defined as ‘educational or learning activities that are mediated by information communication technology (ICT), or web-based applications, where learners or teachers engage with the technology for flexible learning, either exclusively, or in combination with face-to-face approaches’. In the absence of a traditional social presence, TEL facilitates interactive learning and is supported by Web 2.0 technology [13]. Web 2.0 characterises the transformation from the static ‘read-only’ capabilities of the original Web 1.0 into a dynamic ‘read-and-write’ participatory media. This has generated a new paradigm for teaching and learners’ participatory activity by offering interconnectivity, content creation and remixing, and interactivity that accommodates learners’ creative practices [14]. Web 2.0 tools include blogs, wikis, and social networking platforms, and they share a capacity for social cohesion and the social construction of new knowledge [15].

TEL in health care settings can diminish traditional, logistical barriers to learning and offers individualised, tailored, point-of-care learning to meet the multiple needs of professional learners from various practice disciplines [16]. Barriers include time loss due to device functionality, inaptitude with a particular device, and lack of social contact compared with face-to-face learning. Low-level digital literacy can also restrict learning with technology [17]. Digital literacy, in the health care context, defines the ability to learn, work, and develop effectively in a digital workplace and society [18]. Indeterminate expressions of digital literacy can generate ambiguity and misconceptions for educators who are involved in the design of TEL [19]. Digital competence is often assumed despite there being varying levels of aptitude amongst learners and disparities are compounded by an increasing diversity of new technologies [20]. An important distinction is situated between a learners’ technological skill in social and entertainment activity and their intellectual proficiency in using technology effectively for learning [21, 22].

The Technology Acceptance Model can be helpful to explain technology use and acceptance [23]. It is based on the ‘theory of reasoned action’ [24], as a predictive model, that posits the subjective perceived usefulness (PU) and perceived ease of use (PEU) mediating the relationship between external variables and the behavioural intention, or likelihood, of using technology [25]. Teaching with technology is dependent on the integration of traditional approaches and the dynamic interactions between educational content, pedagogy, and the technology. These constructs, and their interactions, are conceptualised in a holistic and context-specific technological pedagogical, and content knowledge (TPACK) [26]. TPACK establishes an ecological perspective when teaching with technology so that the technology is not regarded as being merely supplementary. Barriers to teaching with technology include extrinsic factors such as equipment, time, training, and support. The intrinsic factors are less tangible and include an educators’ pedagogical and technical beliefs [27].

TEL is optimised in its capacity to deliver participatory and activity-centred learning that incorporates strategies to promote purposeful virtual dialogue between learners, and with teachers, by incorporating appropriate design features [28]. The design processes and educational theories that underpin TEL are important determinants when evaluating the effectiveness of interventions [29]. Kirkpatrick’s Four-Level Model is a widely cited framework for evaluating educational and training interventions [30]. It is a four-level model that considers learners’ *reactions* to the training; *learning* gains as knowledge, skills, and attitudinal change; practice-based *behaviour* changes following training; and the wider *results* occurring because of the training. It is subject to criticism for being simplistic and for its assumptions of hierarchy, causality, and inter-correlation between levels [31]. Despite this, the simplicity of the method is a strength [32]; however, it is considered sub-optimal for TEL evaluations [33].

**Fig. 1:**
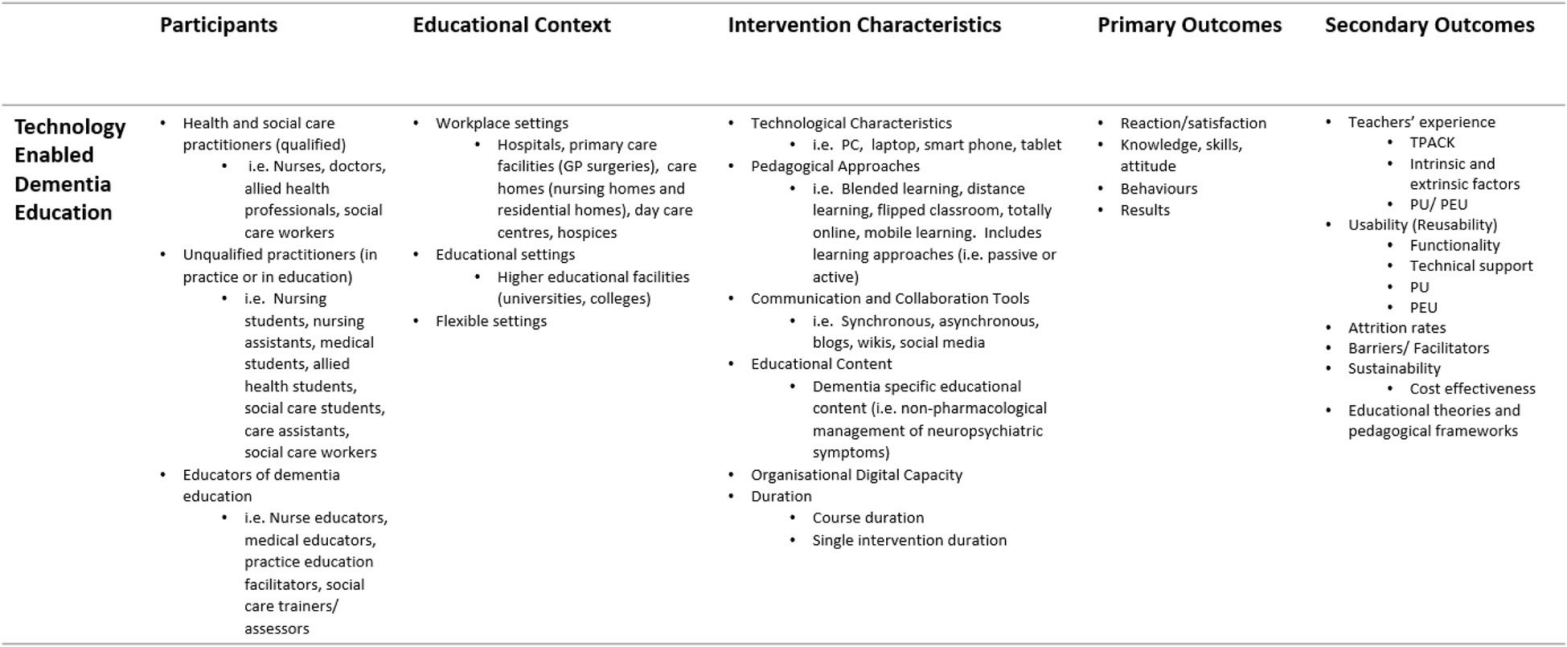
System-based logic model. Adapted from Rohwer et. al. (2017)

**Fig. 2:**
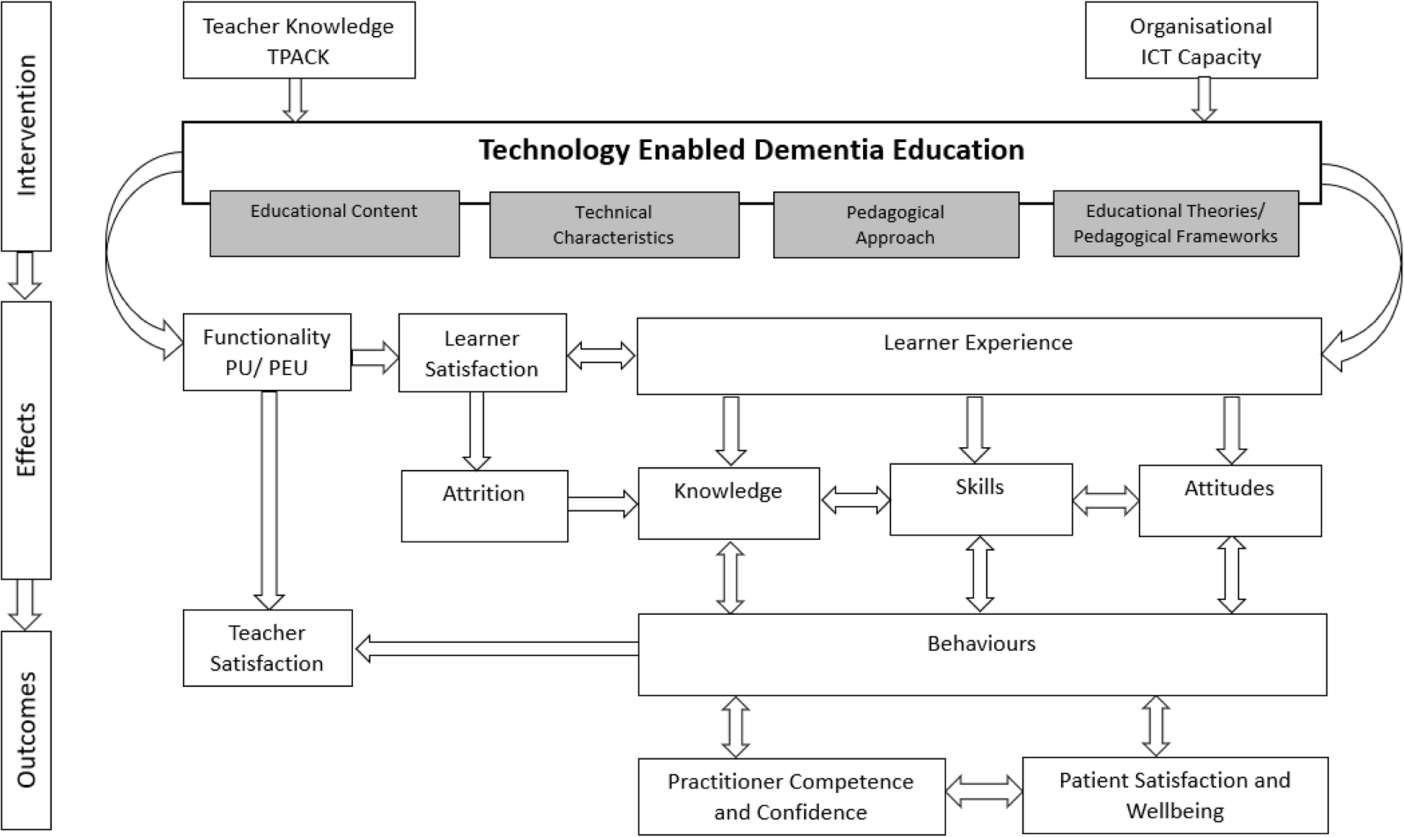
Process-based logic model. Adapted from Rohwer et al (2017)

A review on the effectiveness of TEDE requires capacity to consider substantial functional and technological heterogeneity, supporting content and activities in theory-based dementia education. One way to conceptualise complexity is by employing logic models to unpack intervention characteristics for transparency across the intervention variables and multiple outcomes [34] (Fig. 1). Like this, the contribution of the educators’ TPACK and the organisational digital capacity can be seen to support TEDE in conveying the appropriate content, pedagogy, and technical characteristics, ideally underpinned by educational theories and pedagogical frameworks. The functionality, PU, and PEU of the technology may be evaluated independently but also influence user satisfaction, affecting the knowledge, skills, and attitudes derived from the learners’ overall experience. Learner gains can be viewed as determinants of improved practitioner, patient, and broader organisational outcomes (Fig. 2).

The aim of the proposed review is to establish the characteristics and effectiveness of TEDE for HSCPs by critically appraising and synthesising the available evidence. The research questions are as follows: What are the pedagogical and technological characteristics of TEDE for HSCPs? Is TEDE an effective approach to dementia education for HSCPs?

## Methods

The design and methodology of this review protocol were informed using the Preferred Reporting Items for Systematic Review and Meta-Analysis Protocols (PRISMA-P) guidelines and checklist [35] (see Additional file 1).

### Population

We will incorporate papers that report on data sourced from research participants including all qualified and unqualified HSCPs in either practice or education including educators and instructors.

### Intervention

We will consider all papers that report on interventions of technology-enabled approaches to dementia education. The interventions will include a TEDE approach not limited to online learning, e-learning, web-based learning, distance learning, blended learning, and mobile learning. Where studies report a hybrid approach, the effectiveness of the combined approach will be evaluated. All single interventions, modules, and online courses will be included. The learning approach will be considered whether it is passive (reading, watching videos) or active (interactive, multimedia) as will discernment of any intentional opportunities for reflection from engagement with TEDE resources. Interventions from studies published after 2005 will be included to capture those studies that reflect the technological opportunities since Web 2.0 [36, 37].

### Comparator

Comparators will include usual practice, traditional learning methods, alternative pedagogical approaches, or differing communication and collaboration tools (i.e. synchronous approaches versus asynchronous or social media versus traditional e-learning). Studies that do not involve a comparator will also be included.

### Outcomes

We will report on the educational content, pedagogical approach, and technological specifications.

*Primary outcomes*:

- The effectiveness of TEDE
  - Reaction/satisfaction
  - Knowledge, skills, attitude
  - Behaviours
  - Results

*Secondary outcomes*:

- Experiences, reactions, or satisfaction of educators with TEDE
- The usability or reusability of TEDE
  - Functionality
  - Technical support
  - PU
  - PEU
- The cost-effectiveness of TEDE
- Barriers and facilitators to TEDE
- Attrition rates in TEDE
- Educational theories or pedagogical frameworks that inform TEDE

Outcomes that describe the effectiveness of TEDE are likely to be derived from randomised controlled trials (RCTs), non-randomised studies, or surveys that use numerical data. The validity and reliability of the evaluation instruments used within quantitative studies will be considered. Outcomes such as stakeholders’ perspectives are likely to be derived from qualitative research designs that use narrative data.

### Study design

We will include quantitative, qualitative, and mixed method studies that report on the effectiveness or user perceptions of TEDE.

#### Inclusion criteria

- Studies of adult learners aged > 16 years
- Studies published after 2005
- Quantitative, qualitative, or mixed method studies evaluating TEDE involving HSCPs or health and social care students or educators
- Studies of TEDE in workplace or higher educational settings (including studies where interventions originate from these settings but are completed externally, i.e. in distance learning)
- Studies that do or do not include a description of educational theories or pedagogical frameworks that inform the interventions

#### Exclusion criteria

- Systematic literature reviews and studies or articles with indeterminate or insubstantial research design (including concept papers, discussion papers, theoretical papers, proposals, protocols, editorials, letters, or comments)
- Studies not in the English language
- Pilot or feasibility studies that report on measures of suitability for implementation of a TEDE intervention and do not evaluate a TEDE process
- Books/book chapters
- Studies of educational interventions for informal carers of people living with dementia
- Studies of interventions related to dementia education (i.e. interventions for delirium education)
- Studies that combine interventions for formal and informal carers unless formal carer (HSCPs) outcomes are explicitly reported
- Studies of massively open online courses unless professional engagement is specifically evaluated (i.e. participants from higher education or HSCPs)
- Decision support interventions
- Studies involving DVD or video unless delivered in an online format
- Studies of telephonic educational interventions

### Search strategy

Literature searches will be carried out in MEDLINE (OVID interface), CINAHL Complete (EBSCO interface), ERIC (EBSCO interface), PsycINFO (EBSCO interface), PubMed, Web of Science Core Collection, OVID Nursing Database, and SCOPUS. An initial search was conducted in November 2018; however, the search will be updated before the preparation of the final report so to identify any new studies since the initial search. The International Prospective Register of Systematic Reviews (PROSPERO), Cochrane Database of Systematic Reviews, Campbell Collaboration Online Library, and Ethos database of doctoral thesis will be searched to ensure that comparable works do not exist and are not in progress.

A combination of subject headings and keywords will be included in the search strategy. Whilst all attempts will be made to apply the keywords uniformly throughout all databases, subject headings will be dependent on database specific thesaurus and subject term mapping. Other variances between databases will result from truncation rules and database specific preferences. Subject headings will be included when they are available and closely match the keywords chosen to describe the concepts of dementia, education, or TEL. Keywords were developed a priori and by considering relevant synonyms and concepts in consultation with the review team. To optimise the relevance of results, the ‘explode’ function will be used on subject headings only if all narrower terms are considered relevant or are included as keywords. Any narrower terms that match keywords will be included as independent subject headings.

A combination of subject terms and keywords will be used for dementia. These will be based on common subtypes of dementia [5]. Multi-infarct dementia is included as the most common form of vascular dementia [38]. The keyword ‘education’ will be consistently applied with an unexploded ‘education’ subject heading, where the subject heading is available. This is considered an optimal approach to reducing irrelevant results from a diverse array of educational subheadings existing within educational subject headings. Subject headings and keywords for TEL will reflect a technological approach to education, learning, or training. This will include absolute and partial approaches, for instance, online learning is an absolute approach where blended learning may involve partial use of technology. Technological devices or computer applications will not be included, as these are diverse, numerous, and continually evolving. Instead, terms of ICT that relate to learning or education will be included.

Population characteristics will be identified during title and abstract screening to enable comprehensive identification of various HSCPs, including students and relevant stakeholders. This strategy will reduce omissions through unnecessary database filtering. The full search strategy is presented in Additional file 2.

### Reference management

The titles and abstracts of identified studies will be downloaded from bibliographic databases into RefWorks and duplicate studies will be removed. One reviewer will then screen the titles and abstracts of the studies based on the eligibility criteria. Two other reviewers will screen 10% of the total titles and abstracts, by each screening 5%. If there is any disagreement in suitability for inclusion, a third reviewer will provide arbitration. The next stage will involve a closer scrutiny of full texts considered eligible for inclusion. One reviewer will review the full texts and studies not meeting the eligibility criteria will be removed. A supplementary Microsoft Excel spreadsheet will be created for supplementary reference management and will include study eligibility status, rationale for inclusions/exclusions, and information on locating studies. The database will be made available on a file sharing platform, i.e. Microsoft SharePoint, only when all reviewers have completed the title and abstract screening. One member of the review team will screen the reference lists of all eligible studies for additional studies that satisfy the inclusion and exclusion criteria. The search process will be charted in a Preferred Reporting Items for Systematic Review and Meta-Analysis (PRISMA) flowchart, and the PRISMA checklist will be applied for optimal reporting in the broader review [39].

### Data extraction

The data extraction stage will involve transcribing the relevant information that is reported in primary studies onto a standard form that will be developed in a format that is specific to the review question [40]. Separate data extraction forms will be developed to capture quantitative or qualitative data. Quantitative forms will include study characteristics (citation, author details, study design, aims, country, ethics, participant characteristics, and participant demographics); methods (results of quality assessment, sampling approach, data collection, and data analysis methods); intervention characteristics (educational content, technical characteristics, pedagogical specifications, and comparator or control group characteristics); and outcome data (learner satisfaction, knowledge, skills, attitudes, behaviours, results, educator experience, functionality, technical support, usability, cost-effectiveness, and attrition data). Quantitative data will include sample sizes and values of statistical significance and/or confidence intervals.

Qualitative data extraction forms will include bibliographic information (citation, author details, country, ethics, participant characteristics, and demographics); methods (theoretical and epistemological perspectives, qualitative method, data analysis technique, sampling approach, and results of quality assessment); aims (including the research question); and intervention characteristics (educational content, technical characteristics, and pedagogical specifications). Qualitative data will also be included that relates to learner satisfaction, knowledge, skills, attitudes, behaviours, results, educator/instructor experiences, expressions of usability/reusability, and commentary on educational theories or pedagogical frameworks.

Two authors will pilot-test the data extraction tools on one, randomly selected, quantitative study and one qualitative study. Subsequently, one reviewer will extract the remaining data. The data extraction tool will be developed digitally and stored on a file sharing system, i.e. Microsoft SharePoint, and will be subject to ‘spot checking’ by the review team.

### Dealing with missing data

Where data is missing, or discrepancies exist within the data, and the data is considered relevant, one reviewer will attempt to request it from study authors using a maximum of two emails. Where data appears ambiguous, or not obviously relevant for inclusion, this data will be flagged for discussion between two reviewers. Disagreement will be arbitrated by a third reviewer. Where data is not included and unavailable, a discussion on the impact of missing data will be provided.

### Quality assessment

The Mixed Methods Appraisal Tool (MMAT) is designed for the appraisal of qualitative studies, RCTs, non-randomised quantitative studies, quantitative descriptive studies, and mixed method research studies. Due to the anticipated heterogeneity of study designs that will be included in the review, the MMAT was considered relevant. Further, its specific design intention for mixed study appraisal encourages an inclusive approach to quality assessment. It has been content validated and pilot-tested for reliability [41]. The MMAT criteria are scored on a nominal scale (yes, no, can’t tell) which allows for detailed presentations of studies of higher, lower, or indeterminate methodological quality. The MMAT comprises a checklist and detailed explanations for methodological quality criteria specific to each study design [42]. One reviewer will assess the methodological quality of eligible studies using the current MMAT (2018 version) and record the nominal values of quality assessment and describe justification for decisions. The quality appraisal documents will be maintained digitally and uploaded onto a file sharing system, i.e. Microsoft SharePoint, for accessibility to the review team where they will be subject to spot checks.

### Data synthesis

**Fig. 3:**
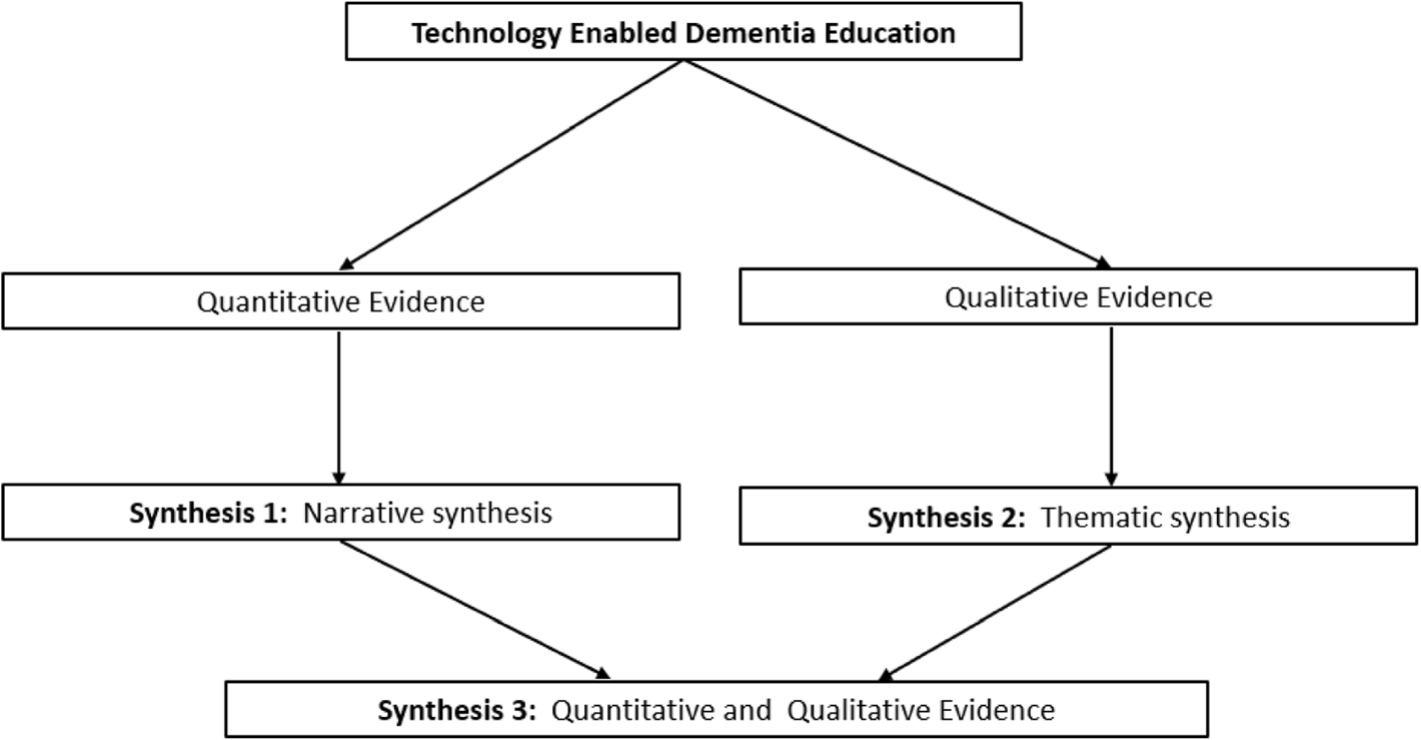
Proposed method for including quantitative and qualitative data. Adapted from Harden (2010)

Data synthesis involving quantitative and qualitative research requires that researchers consider ways to handle the methodological diversity within and between qualitative and quantitative studies. A segregated approach to data synthesis recognises the binary distinction between quantitative and qualitative research. This approach will allow quantitative data to be synthesised independently of qualitative data, but with capacity to compliment, confirm, or refute evidence from the divergent paradigm [43]. This approach will be beneficial when considering quantifiable learner gains with qualitative expressions of learner satisfaction. A proposed method to combining quantitative and qualitative findings is illustrated (Fig. 3) [44].

Due to anticipated interventional and methodological heterogeneity between studies, it is unlikely that the metrics arising from diverse sources of quantitative data will be amenable to meta-analysis. Findings will therefore be reported in narrative synthesis by tabulating the outcome data. This will include effect sizes and reports on statistically and non-statistically significant results. The sample size and direction of effect will also be included so that precision and weighting can be considered when quantifying effectiveness at varying significance levels. Additionally, quality assessment ratings will be incorporated with the synthesis so that outcomes can be considered in relation to methodological quality and not merely on any evidence of effect.

A thematic synthesis of qualitative data will be conducted should the data be amenable. Textual data, describing the views of participants, and key findings by the researcher will be identified, on an inductive basis, resulting in descriptive themes that will be further developed into analytical themes [45]. These analytical themes will then be interpreted to produce rich and deep understanding of the phenomena with capacity to complement, confirm, or refute any evidence from the quantitative data. A comprehensive and transparent account describing the conduct of the thematic synthesis will be provided.

A textual interpretation of all findings will be presented to form an overall picture of the current knowledge. To uphold internal validity and transparency in the methods of narrative synthesis, guidance will be integrated from the UK’s Economic and Social Research Council (ESRC) [46].

### Analysis of subgroups

Subgroup analysis can be helpful to explore the impact of potential effect modifiers on the effects of an intervention and may be helpful to understand which TEDE interventions works best, and for whom [47]. Therefore, interventions of shared learner characteristics will be reported in subgroups so to discern the effectiveness of TEDE within specific learner groups. There is anticipated substantial interventional heterogeneity; therefore, shared technological or pedagogical characteristics will be explored in subgroups should the data be amenable. The newest and emerging TEDE approaches will also be investigated in subgroups, with particular relevance to their technological and pedagogical specifications, thereby considering effectiveness in the context of the most recent technological advances. The methods for implementing subgroups in the review will be informed by guidance from the ESRC [46].

### Overall quality of the evidence

The outcomes from quantitative data will be defined in high-, moderate-, low-, and/or very low-quality categories using the GRADE approach [48, 49]. As the quantitative evidence will be presented in narrative synthesis, the certainty of the evidence will be presented in the absence of a single (combined) estimate of effect [50]. The GRADE-CERQual approach will inform the confidence of the findings from qualitative evidence [51, 52].

## Discussion

This protocol aims to demonstrate the planned methodological and analytical approaches that will inform a systematic review of TEDE. The design is strengthened by adhering to PRISMA-P guidance for increased clarity, transparency, and future reproducibility [35]. Pre-registering the protocol with PROSPERO reduces the risk of reporting bias in a completed review [53]. The research questions have been clearly articulated and broken down into searchable keywords [54]. Therefore, a comprehensive search strategy can be applied to several scientific, health-related, biomedical, and educational databases. Whilst it may be inevitable that not all relevant studies can be sourced for a review, the study selection process is optimised by searching the reference lists of all eligible studies for additional studies also meeting the eligibility criteria.

Every attempt has been made to provide a comprehensive inclusion and exclusion criteria, although this cannot guarantee against elements of subjectivity and human error in the study selection process [55]. Single reviewer title and abstract screening is a limitation of the protocol design but is necessary due to the available resources and is mitigated by second reviewers screening a total of 10% of potentially relevant studies. A similar limitation results from single reviewer quality assessment; therefore, the use of a reliability tested and validated tool (MMAT) will add rigour to this process and help to reduce bias from the individual studies. A single reviewer will also complete data extraction; however, the data extraction tools will be piloted with two reviewers. A process of spot checking has been implemented for data extraction and quality assessment in order to further mitigate against error.

The participants include a wide range of HSCPs from various occupational and educational levels which are considered to be important influences on learning outcomes. The effectiveness of TEDE is therefore established in a heterogeneous population, and it is necessary that this diversity is acknowledged; therefore, specific learner characteristics are intended to be investigated, in relation to their outcomes, in a subgroup analysis. This will be particularly relevant when reporting on the overall effectiveness of TEDE. The heterogeneity of interventional characteristics is somewhat expected in a review of TEDE, considering the various technological and pedagogical approaches. A narrative synthesis is therefore more appropriate than statistical methods of analysis and the avoidance of bias is the main factor to a rigorous synthesis of data. Advanced specification of the intended methods, including guidance from ESRC, will promote rigour and transparency [47].

This protocol is intended to inform the development of a completed review which will aim to support educators, practitioners, and other stakeholders in the design and implementation of TEDE programmes for HSCPs. It is also intended to advance the current academic literature and potentiate further research into TEDE at a time of unprecedented demographic and technological transition.

## Supplementary information

**Additional file 1.** PRISMA-P (Preferred Reporting Items for Systematic review and Meta-Analysis Protocols) 2015 checklist: recommended items to address in a systematic review protocol.

**Additional file 2.** Search Strategy.

## Data Availability

Not applicable.

## Abbreviations

ESRC: Economic and Social Research Council
HSCP: Health and social care practitioner
ICT: Information communication technology
MMAT: Mixed Methods Appraisal Tool
PEU: Perceived ease of use
PRISMA: Preferred Reporting Items for Systematic Review and Meta-Analysis
PRISMA-P: Preferred Reporting Items for Systematic Review and Meta-Analysis Protocols
PROSPERO: International Prospective Register of Systematic Reviews
PU: Perceived usefulness
RCT: Randomised controlled trial
TEDE: Technology-enabled dementia education
TEL: Technology-enabled learning
TPACK: Technological pedagogical and content knowledge
