# Supplementary material for: The characteristics of effective technology-enabled dementia education for health and social care practitioners: protocol for a mixed studies systematic review
Source: Syst Rev. 2019 Dec 6;8:316. doi: 10.1186/s13643-019-1212-4 (PMC6896733; doi:10.1186/s13643-019-1212-4)
Supplement: Supplementary file 2 — Additional file 2. Search Strategy. [file 13643_2019_1212_MOESM2_ESM.docx]

Additional file 2. Search Strategy

**OVID MEDLINE**

1. DEMENTIA/

2. Alzheimer Disease/

3. Dementia, Multi-Infarct/

4. Dementia, Vascular/

5. Frontotemporal Dementia/

6. Lewy Body Disease/

7. (dement* or alzheimer* or fronto* dement* or lewy bod* dement* or dement* with lewy bod* or ((multiinfarct or multi-infarct) and dement*) or vascular dement*).af.

8. 1 or 2 or 3 or 4 or 5 or 6 or 7

9. EDUCATION/

10. education.af.

11. 9 or 10

12. Computer-Assisted Instruction/

13. Education, Distance/

14. (technol* en* learn* or technol* en* educat* or technol* based learn* or technol* based educat*).af.

15. (e-learn* or elearn* or electronic learn*).af.

16. (online learn* or online educat* or online cours* or online train* or online support* or online instruct* or online CPD or online profession* develop*).af.

17. (on-line learn* or on-line educat* or on-line cours* or on-line train* or on-line support* or on-line instruct* or on-line CPD or on-line profession* develop*).af.

18. (internet adj3 (learn* or educat*)).af.

19. (web-based learn* or web-based educat* or web-based instruct*).af.

20. (web 2* adj3 (educat* or learn*)).af.

21. (digital learn* or digital educat*).af.

22. (m-learn* or mlearn* or m-educat* or meducat* or mobile learn* or mobile educat*).af

23. (distance learn* or distance educat*).af.

24. (hybrid learn* or hybrid educat*).af.

25. (blended learn* or blended educat*).af.

26. (ICT adj3 (learn* or educat*)).af.

27. ("information communication technology" adj3 (learn* or educat*)).af.

28. (multimedia learn* or multi-media learn* or multimedia instruct* or multi-media instruct*).af.

29. (virtual learn* or virtual educat*).af.

30. (interactive learn* or interactive educat*).af.

31. (computer* adj3 (learn* or educat* or instruct* or train*)).af.

32. 12 or 13 or 14 or 15 or 16 or 17 or 18 or 19 or 20 or 21 or 22 or 23 or 24 or 25 or 26 or 27 or 28 or 29 or 30 or 31

33. 8 and 11 and 32

**OVID NURSING DATABASE**

1. dementia/

2. alzheimer's disease/

3. multi-infarct dementia/

4. Vascular Dementia/

5. (dement* or alzheimer* or fronto* dement* or lewy bod* dement* or dement* with lewy bod* or ((multiinfarct or multi-infarct) and dement*) or vascular dement*).af.

6. 1 or 2 or 3 or 4 or 5

7. Education/

8. education.af.

9. 7 or 8

10. computer-assisted instruction/

11. (technol* en* learn* or technol* en* educat* or technol* based learn* or technol* based educat*).af.

12. (e-learn* or elearn* or electronic learn*).af.

13. (online learn* or online educat* or online cours* or online train* or online support* or online instruct* or online CPD or online profession* develop*).af.

14. (on-line learn* or on-line educat* or on-line cours* or on-line train* or on-line support* or on-line instruct* or on-line CPD or on-line profession* develop*).af.

15. (internet adj3 (learn* or educat*)).af.

16. (web-based learn* or web-based educat* or web-based instruct*).af.

17. (web 2* adj3 (educat* or learn*)).af.

18. (digital learn* or digital educat*).af.

19. (m-learn* or mlearn* or m-educat* or meducat* or mobile learn* or mobile educat*).af.

20. (distance learn* or distance educat*).af.

21. (hybrid learn* or hybrid educat*).af.

22. (blended learn* or blended educat*).af.

23. (ICT adj3 (learn* or educat*)).af.

24. ("information communication technology" adj3 (learn* or educat*)).af.

25. (multimedia learn* or multi-media learn* or multimedia instruct* or multi-media instruct*).af.

26. (virtual learn* or virtual educat*).af.

27. (interactive learn* or interactive educat*).af.

28. (computer* adj3 (learn* or educat* or instruct* or train*)).af.

29. 10 or 11 or 12 or 13 or 14 or 15 or 16 or 17 or 18 or 19 or 20 or 21 or 22 or 23 or 24 or 25 or 26 or 27 or 28

30. 6 and 9 and 29

**EBSCO CINAHL**

S32 S8 AND S11 AND S31

S31 S12 OR S13 OR S14 OR S15 OR S16 OR S17 OR S18 OR S19 OR S20 OR S21 OR S22 OR S23 OR S24 OR S25 OR S26 OR S27 OR S28 OR S29 OR S30

S30 (computer* N3 (learn* or educat* or instruct* or train*))

S29 (interactive (learn* or educat*))

S28 (virtual (learn* or educat*))

S27 (multimedia or multi-media) and (learn* or instruct*)

S26 ("information communication technology" N3 (learn* or educat*))

S25 (ICT N3 (learn* or educat*))

S24 (blended (learn* or educat*))

S23 (hybrid (learn* or educat*))

S22 (distance (learn* or educat*))

S21 (m-learn* or mlearn* or m-educat* or meducat* or mobile learn* or mobile educat*)

S20 (digital (learn* or educat*))

S19 (web 2* N3 (educat* or learn*))

S18 (web-based (learn* or educat* or instruct*))

S17 (internet N3 (learn* or educat*))

S16 (on-line (learn* or educat* or cours* or train* or support* or instruct* or CPD or profession* develop*))

S15 (online (learn* or educat* or cours* or train* or support* or instruct* or CPD or profession* develop*))

S14 (e-learn* or elearn* or electronic learn*)

S13 (technol* en* learn* or technol* en* educat* or technol* based learn* or technol* based educat*

S12 (MH "Computer Assisted Instruction")

S11 S9 OR S10

S10 education

S9 (MH "Education")

S8 S1 OR S2 OR S3 OR S4 OR S5 OR S6 OR S7

S7 (dement* or alzheimer* or fronto* dement* or lewy bod* dement* or dement* with lewy bod* or ((multiinfarct or multi-infarct) and dement*) or vascular dement*)

S6 (MH "Lewy Body Disease")

S5 (MH "Frontotemporal Dementia")

S4 (MH "Dementia, Multi-Infarct")

S3 (MH "Dementia, Vascular")

S2 (MH "Alzheimer's Disease")

S1 (MH "Dementia")

**EBSCO PsycINFO**

S33 S7 AND S10 AND S32

S32 S11 OR S12 OR S13 OR S14 OR S15 OR S16 OR S17 OR S18 OR S19 OR S20 OR S21 OR S22 OR S23 OR S24 OR S25 OR S26 OR S27 OR S28 OR S29 OR S30 OR S31

S31 (computer* N3 (learn* or educat* or instruct* or train*))

S30 (interactive (learn* or educat*))

S29 (virtual (learn* or educat*))

S28 (multimedia or multi-media) and (learn* or instruct*)

S27 ("information communication technology" N3 (learn* or educat*))

S26 (ICT N3 (learn* or educat*))

S25 (blended (learn* or educat*))

S24 (hybrid (learn* or educat*))

S23 (distance (learn* or educat*))

S22 (m-learn* or mlearn* or m-educat* or meducat* or mobile learn* or mobile educat*)

S21 (digital (learn* or educat*))

S20 (web 2* N3 (educat* or learn*))

S19 (web-based (learn* or educat* or instruct*))

S18 (internet N3 (learn* or educat*))

S17 (on-line (learn* or educat* or cours* or train* or support* or instruct* or CPD or profession* develop*))

S16 (online (learn* or educat* or cours* or train* or support* or instruct* or CPD or profession* develop*))

S15 (e-learn* or elearn* or electronic learn*)

S14 (technol* en* learn* or technol* en* educat* or technol* based learn* or technol* based educat*)

S13 DE "Electronic Learning"

S12 DE "Distance Education"

S11 DE "Computer Assisted Instruction"

S10 S8 OR S9

S9 education

S8 DE "Education"

S7 S1 OR S2 OR S3 OR S4 OR S5 OR S6

S6 (dement* or alzheimer* or fronto* dement* or lewy bod* dement* or dement* with lewy bod* or ((multiinfarct or multi-infarct) and dement*) or vascular dement*)

S5 DE "Semantic Dementia"^1^

S4 DE "Dementia with Lewy Bodies"

S3 DE "Vascular Dementia"^2^

S2 DE "Alzheimer's Disease"

S1 DE "Dementia"

1. *Semantic dementia is used for frontotemporal lobe dementia as recommended in PsycInfo thesaurus. Frontotemporal lobe dementia is included as a relevant keyword for consistency between other databases.*
2. *Vascular dementia includes multi infarct dementia in the PsycInfo thesaurus. Multi infarct dementia is included as an independent keyword for consistency between other databases.*

**EBSCO ERIC**

S34 S4 AND S7 AND S33

S33 S8 OR S9 OR S10 OR S11 OR S12 OR S13 OR S14 OR S15 OR S16 OR S17 OR S18 OR S19 OR S20 OR S21 OR S22 OR S23 OR S24 OR S25 OR S26 OR S27 OR S28 OR S29 OR S30 OR S31 OR S32

S32 (computer* N3 (learn* or educat* or instruct* or train*))

S31 (interactive (learn* or educat*))

S30 (virtual (learn* or educat*))

S29 (multimedia or multi-media) and (learn* or instruct*)

S28 ("information communication technology" N3 (learn* or educat*))

S27 ICT N3 (learn* or educat*))

S26 (blended (learn* or educat*))

S25 (hybrid (learn* or educat*))

S24 (distance (learn* or educat*))

S23 (m-learn* or mlearn* or m-educat* or meducat* or mobile learn* or mobile educat

S22 (digital (learn* or educat*))

S21 (web 2* N3 (educat* or learn*))

S20 (web-based (learn* or educat* or instruct*))

S19 (internet N3 (learn* or educat*))

S18 (on-line (learn* or educat* or cours* or train* or support* or instruct* or CPD or profession* develop*))

S17 (online (learn* or educat* or cours* or train* or support* or instruct* or CPD or profession* develop*))

S16 (e-learn* or elearn* or electronic learn*)

S15 (technol* en* learn* or technol* en* educat* or technol* based learn* or technol* based educat*)

S14 DE "Blended Learning"

S13 DE "Electronic Learning"

S12 DE "Distance Education"

S11 DE "Multimedia Instruction"

S10 DE "Web Based Instruction"

S9 DE "Online Courses"

S8 DE "Computer Assisted Instruction"

S7 S5 OR S6

S6 education

S5 DE "Education"

S4 S1 OR S2 OR S3

S3 (dement* or alzheimer* or fronto* dement* or lewy bod* dement* or dement* with lewy bod* or ((multiinfarct or multi-infarct) and dement*) or vascular dement*)

S2 DE "Alzheimers Disease"

S1 DE "Dementia"

**Web of Science**

#22 #21 AND #2 AND #1

#21 #20 OR #19 OR #18 OR #17 OR #16 OR #15 OR #14 OR #13 OR #12 OR #11 OR #10 OR #9 OR #8 OR #7 OR #6 OR #5 OR #4 OR #3

#20 TS= (computer* NEAR/3 (learn* or educat* or instruct* or train*))

#19 TS= ("interactive learn*" or "interactive educat*")

#18 TS= ("virtual learn*" or "virtual educat*")

#17 TS= ("multimedia learn*" or "multi-media learn*" or "multimedia instruct*" or "multi-media instruct*")

#16 TS= ("information communication technology" NEAR/3 (learn* or educat*))

#15 TS= (ICT NEAR/3 (learn* or educat*))

#14 TS= ("blended learn*" or "blended educat*")

#13 TS= ("hybrid learn*" or "hybrid educat*")

#12 TS= ("distance learn*" or "distance educat*")

#11 TS= ("m-learn*" or "mlearn*" or "m-educat*" or "meducat*" or "mobile learn*" or "mobile educat*")

#10 TS= ("digital learn*" or "digital educat*")

#9 TS= (web 2 NEAR/3 (educat* or learn*))

#8 TS= ("web-based learn*" or "web-based educat*" or "web-based instruct*")

#7 TS= (internet NEAR/3 (learn* or educat*))

#6 TS= ("on-line learn*" or "on-line educat*" or "on-line cours*" or "on-line train*" or "on-line support*" or "on-line instruct*" or "on-line CPD" or "on-line profession* develop*")

#5 TS= ("online learn*" or "online educat*" or "online cours*" or "online train*" or "online support*" or "online instruct*" or "online CPD" or "online profession* develop*")

#4 TS= ("e-learn*" or "elearn*" or "electronic learn*")

#3 TS= ("technol* en* learn*" or "technol* en educat*" or "technol* based learn*" or "technol* based educat*")

#2 TS= (education)

#1 TS= ((dement* or alz* or fronto* dement* or lewy bod* dement* or dement* with lewy bod* or ((multiinfarct or multi-infarct) and dement*) or vascular dement*))

**SCOPUS**

TITLE-ABS-KEY ( dementia* OR alzheimer* OR "fronto* dement*" OR "lewy bod* dement*" OR "dement* with lewy bod*" OR "multiinfarct dement*" OR "multi-infarct dement*" OR "vascular dement*" ) AND TITLE-ABS-KEY ( education ) AND TITLE-ABS-KEY ( "technol* en* learn*" OR "technol* en* educat*" OR "technol* based learn*" OR "technol* based educat*" OR "e-learn*" OR elearn* OR "electronic learn*" OR "online learn*" OR "online educat*" OR "online cours*" OR "online train*" OR "online support*" OR "online instruct*" OR "online CPD" OR "online profession* develop*" OR "on-line learn*" OR "on-line educat*" OR "on-line cours*" OR "on-line train*" OR "on-line support*" OR "on-line instruct*" OR "on-line CPD" OR "on-line profession* develop*" OR ( internet W/3 learn* ) OR ( internet W/3 educat* ) OR "web-based learn*" OR "web-based educat*" OR "web-based instruct*" OR ( "web 2*" W/3 learn* ) OR ( "web 2*" W/3 educat* ) OR "digital learn*" OR "digital educat*" OR m-learn* OR mlearn* OR m-educat* OR meducat* OR "mobile learn*" OR "mobile educat*" OR "distance learn*" OR "distance educat*" OR "hybrid learn*" OR "hybrid educat*" OR "blended learn*" OR "blended educat*" OR ( ict W/3 learn* ) OR ( ict W/3 educat* ) OR ( "information communication technology" W/3 learn* ) OR ( "information communication technology" W/3 educat* ) OR "multimedia learn*" OR "multi-media learn*" OR "multimedia instruct*" OR "multi-media instruct*" OR "virtual learn*" OR "virtual educat*" OR "interactive learn*" OR "interactive educat*" OR ( computer* W/3 learn* ) OR ( computer* W/3 educat* ) OR ( computer* W/3 instruct* ) OR ( computer* W/3 train* ) ) AND ( LIMIT-TO ( EXACTKEYWORD , "Dementia" ) OR LIMIT-TO ( EXACTKEYWORD , "Education" ) )

**PUBMED Demetria**

Search ((((((((dementia) OR alzheimer's disease) OR frontotemporal dementia) OR lewy body dementia) OR vascular dementia) OR multi infarct dementia)) AND education) AND (((((((((((((((((((((((((((((((((((((((((((((((((((((((((("technology enabled learning") OR "technology enhanced learning") OR "technology enhanced education") OR "technology based learning") OR "technology based education") OR e-learning) OR "electronic learning") OR "online learning") OR "online education") OR "online course") OR "online training") OR "online support") OR "online instruction") OR (online CPD OR online AND "professional development")) OR "on-line learning") OR "on-line education") OR "on-line course") OR "on-line training") OR "on-line support") OR "on-line instruction") OR (on-line CPD OR on-line AND "professional development")) OR internet learn*) OR internet educat*) OR internet based learn*) OR internet based educat*) OR web based learn*) OR web based educat*) OR web based instruct*) OR digital learn*) OR digital educat*) OR ("web 2.0" AND learning)) OR ("web 2.0" AND education)) OR m-learn*) OR mobile learn*) OR "education, distance"[MeSH Terms]) OR "distance education") OR "hybrid learning") OR "hybrid education") OR "blended learning") OR "blended education") OR ("ICT" OR "information communication technology" AND learning)) OR ("ICT" OR "information communication technology" AND education)) OR multimedia learn*) OR multimedia instruct*) OR virtual learn*) OR virtual educat*) OR interactive learn*) OR interactive educat*) OR "computer assisted instruction") OR "computer assisted education") OR "computer assisted learning") OR "computer based instruction") OR "computer based education") OR "computer based learning") OR "computer mediated instruction") OR "computer mediated education") OR "computer mediated learning") OR "distance learning")^1^

^1^ *MeSH terms are included only as indicated; related terms found in PubMed search details were not included. There were no results available for “technology enabled education “at the time of database searching. As no proximity searching feature is available in PubMed, we have added additional concepts for internet learning/ education and computer learning/ education. We have used the Boolean operator AND with web 2.0 learning/ education and information communication technology (ICT) learning/education to compensate for unavailable database filtering and to create consistency between other databases.*
